# Ischemic Lesion Location Based on the ASPECT Score for Risk Assessment of Neurogenic Dysphagia

**DOI:** 10.1007/s00455-020-10204-0

**Published:** 2020-11-06

**Authors:** Sriramya Lapa, Christian Foerch, Oliver C. Singer, Elke Hattingen, Sebastian Luger

**Affiliations:** 1 Department of Neurology, Neurovascular Research Group, Goethe University Hospital Frankfurt, Schleusenweg 2-16, 60528 Frankfurt, Germany; 2 Institute of Neuroradiology, Goethe University, Frankfurt, Germany

**Keywords:** Dysphagia, Swallowing, Stroke, ASPECTS, Pneumonia

## Abstract

Dysphagia is common in patients with middle cerebral artery (MCA) infarctions and associated with malnutrition, pneumonia, and mortality. Besides bedside screening tools, brain imaging findings may help to timely identify patients with swallowing disorders. We investigated whether the *Alberta stroke program early CT score* (ASPECTS) allows for the correlation of distinct ischemic lesion patterns with dysphagia. We prospectively examined 113 consecutive patients with acute MCA infarctions. Fiberoptic endoscopic evaluation of swallowing (FEES) was performed within 24 h after admission for validation of dysphagia. Brain imaging (CT or MRI) was rated for ischemic changes according to the ASPECT score. 62 patients (54.9%) had FEES-proven dysphagia. In left hemispheric strokes, the strongest associations between the ASPECTS sectors and dysphagia were found for the lentiform nucleus (odds ratio 0.113 [CI 0.028–0.433; *p* = 0.001), the insula (0.275 [0.102–0.742]; *p* = 0.011), and the frontal operculum (0.280 [CI 0.094–0.834]; *p* = 0.022). A combination of two or even all three of these sectors together increased relative dysphagia frequency up to 100%. For right hemispheric strokes, only non-significant associations were found which were strongest for the insula region. The distribution of early ischemic changes in the MCA territory according to ASPECTS may be used as risk indicator of neurogenic dysphagia in MCA infarction, particularly when the left hemisphere is affected. However, due to the exploratory nature of this research, external validation studies of these findings are warranted in future.

**Electronic supplementary material:**

The online version of this article (10.1007/s00455-020-10204-0) contains supplementary material, which is available to authorized users.

## Introduction

Dysphagia is common among patients with acute ischemic stroke with a reported incidence of up to 78% [1]. It is associated with an increased rate of in-hospital complications (such as malnutrition and aspiration pneumonia) and with unfavorable long-term functional outcome [2, 3]. Therefore, early identification of patients with post-stroke dysphagia is of utmost importance in order to guide decisions concerning nutrition status, drug administration and necessity of further instrumental diagnostics (e.g., fiberoptic endoscopic evaluation of swallowing and/or videofluoroscopic swallowing study). Consequently, the current standard of stroke care requires that all patients should undergo a standardized dysphagia screening.

Although many different swallowing screening tests, which have been validated in the stroke population, provide acceptable sensitivity, no single test has been accepted as the gold standard yet [4–9]. Hence, there is an urgent need to establish additional clinical parameters, e.g., radiological findings, to raise diagnostic accuracy in identifying dysphagic patients. Individuals with acute infarctions involving brain areas highly related to dysphagia could be targeted for expedited referral to a Speech and Language Pathologist (SLP) and/or instrumental swallowing assessment [10, 11].

Several studies have shown that volume, location, and lateralization of the lesion are associated with dysphagia incidence, severity, and characteristics of swallowing dysfunction [12–17]. While right hemispheric stroke was linked to pharyngeal-stage dysfunction, aspiration, and higher dysphagia frequency, left hemispheric stroke was connected to oral-stage dysfunction and less severe dysphagia [13, 18, 19]. Furthermore, lesions in distinct brain areas such as the pre- and post-central gyri, opercular region, supra-marginal gyrus, and adjacent subcortical white matter tracts were identified to be related to dysphagia, with post-central lesions being especially associated with severe swallowing impairment [13]. Although considered to be a valuable information, the implementation of such radiological findings into clinical routine is challenging.

In this context, the *Alberta stroke program early CT score* (ASPECTS) can provide a tailored remedy: ASPECTS is a 10-point quantitative topographic CT scan score originally designed to quantify the degree of early ischemic changes in the brain parenchyma of patients with middle cerebral artery (MCA) stroke. By dividing the MCA territory in ten segments, it might allow for a standardized correlation between certain brain areas and dysphagia. A particular advantage of ASPECTS is its quick and easy applicability without the need for additional imaging processing steps, while offering simultaneously high reliability and reproducibility [20].

Given that evaluation according to the ASPECTS criteria is routinely performed in acute stroke care, a detailed investigation whether individually affected segments or the sum score might help to identify stroke patients at risk for dysphagia is of high practical importance. Since swallowing is a midline function with bilateral cortical activation, it remains controversial to what extent swallowing function is lateralized [21–23]. Therefore, it would be particularly interesting to know whether differences regarding the association of affected ASPECT segments or the sum score exist between the right and the left hemisphere.

## Methods

### Study Design

113 consecutive patients with acute ischemic stroke in the MCA territory were included in this study. A sub-cohort had been previously analyzed [24]. The study was approved by the ethics committee of the Goethe University Hospital Frankfurt and was conducted according to the principles of the Declaration of Helsinki. All patients, resp. their legal representatives, gave their informed consent prior to their inclusion in the study.

### Patients

Patients were included within 72 h after symptom onset if they had clinical and imaging evidence (computed tomography including CT angiography or magnetic resonance imaging including MR angiography) of MCA infarction. Exclusion criteria were: age younger than 18 years, imaging evidence of intracerebral hemorrhage, imaging evidence of ischemic stroke also in other vascular territories, resolution of symptoms within 24 h (i.e., transitory ischemic attack), impaired level of consciousness, need for immediate orotracheal intubation or neurosurgical interventions. Patients having pre-existing dysphagia or any concomitant disease likely to cause dysphagia were also excluded.

### Dysphagia Assessment

All patients received a full assessment of speech, voice, language, and swallowing function including instrumental swallowing assessment within 24 h after admission. For the purpose of this study fiberoptic endoscopic evaluation of swallowing (FEES) was used to assess dysphagia. It has been shown to be an excellent objective method for a quick, safe, and precise dysphagia assessment in the first days after stroke [1, 25, 26]. FEES can be performed bedside at short notice at the intensive care unit (ICU) and/or stroke unit, enabling evaluation of patients in critical medical conditions, which frequently applies to patients with severe stroke. In a first step, the presence of dysarthria, dysphonia, abnormal volitional cough, abnormal gag reflex, aphasia, and buccofacial apraxia were evaluated [24, 27, 28]. In a second step, all patients underwent FEES to assess the presence of dysphagia (primary endpoint) and to classify dysphagia severity (secondary endpoint). Patients were rated as being dysphagic when one or more of the following signs of swallowing dysfunction were detected during endoscopic swallowing examination: disturbed management of secretions (i.e., pooling or aspiration of saliva), penetration or aspiration of any food consistency, relevant pharyngeal food residue after the swallow, or premature spillage with delayed initiation of the swallowing reflex [29]. Dysphagia severity was rated according to the fiberoptic dysphagia severity scale (FEDSS) with 1 scoring best and 6 being worst. Patients were categorized in mildly dysphagic (1–3) and severely dysphagic [13].

FEES equipment consisted of a 3.1-mm-diameter flexible fiberoptic rhinolaryngoscope (ENF-P4, Olympus, Hamburg Germany), a 150 W light source for endoscopic application (rp-150), a camera (rpCam62, S/N), a color monitor (7′-TFT-EIZO, 1500:1), and a video recorder (1/2” CCD-Kamera, rp Cam62). All examinations were videotaped. FEES procedures were performed from a neurologist and a SLP, both having several years of experience with the diagnostic tool. We followed a standardized FEES protocol [30]. In brief, the examination started with rating the severity of oropharyngeal secretions. Next, different food consistencies were applied to the patient starting with thickened water followed by semisolid (pudding), liquid, and solid (white bread) textures. All food was dyed with blue food coloring for better contrast with pharyngeal and laryngeal mucosa. Each consistency was regularly tested three times, but we refrained from further examinations with the respective texture in case of aspiration without total ejection of the material (PAS 7 and 8).

In addition, patients were evaluated for the occurrence of (aspiration) pneumonia using the same criteria as the ones employed in a previous study [31]: Pneumonia was defined as nosocomial, hospital-acquired pneumonia occurring during hospitalization, at least ≥ 48 h after admission, documented by a physician, and requiring antibiotic treatment.

### Brain Imaging Assessment

Brain images were independently rated by a blinded neuroradiologist (EH) and a blinded neurologist (OCS), both experienced in the evaluation of brain imaging in stroke patients. If ASPECTS differed ≤ 1 point, the neuroradiologist’s judgment was followed. If ASPECTS deviated ≥ 2 points between the two raters (*n* = 22), a joint re-evaluation was performed. The ASPECT score was determined either on the first CT scan with clear visible ischemic changes or, if available, on the first MRI scan (to assess DWI-ASPECTS). Median time interval between hospital admission and the brain imaging used for ASPECTS evaluation was 1 day (IQR 0–4).

In brief, the MCA territory in both hemispheres was divided into 10 regions (see Table 2), each representing 1 point on the 10-point ASPECT score. A single point was subtracted for an area of ischemic changes on CT, respectively, on MRI. On CT scans (*n* = 76 patients), ischemic changes were evaluated according the recommendations of the Alberta stroke program (https://www.aspectsinstroke.com/). On CT scans, ischemic changes were defined as any or all of hypoattenuation of the basal ganglia, loss of insular ribbon (insular ribbon sign), loss of grey–white matter differentiation due to cortical hypodensity, hemispheric sulcus effacement due to cortical swelling. All MRI scans (in *n* = 37 patients) included axial diffusion-weighted images on which the DWI-ASPECT score was evaluated [32]. For DWI sequences, focal ischemia was defined by hyperintense signal.

### Statistical Analysis

The *t*-test (for parametric data), the Mann–Whitney *U* test (for non-parametric data) and the chi-square test (for binary variables) were used to test for differences between patients with and without dysphagia. Odds ratios (OR) for the occurrence of dysphagia (vs. no dysphagia) were calculated for each of the 10 ASPECTS segments, separately for the left and the right hemisphere using individual regression analyses. In addition, a stepwise multivariate regression model was used to identify ASPECTS segments that were independently associated with dysphagia. A second multivariate regression model was used to evaluate if the ASPECT sum score adjusted for age and sex (and NIHSS explorative) was independently associated with dysphagia. A third multivariate regression analysis adjusted for age and sex was performed to analyze potential associations between ASPECT sum scores and the occurrence of pneumonia. A significance level of alpha = 0.05 was chosen for all tests. Data were analyzed using SPSS 19 (IBM Corporation, Somers, N.Y., USA). A *heat map* was created using Office Excel 2017 (Microsoft Corporation, Redmond, USA) to visually display the OR values derived from the individual regression analyses (see above). For doing so the location of each of the 10 ASPECTS segments was manually marked on both hemispheres of the sample CT scan. Subsequently, for each ASPECTS segment, the software tool assigns a color tone from a defined continuous color range according to the given OR values (from 1.0 to 0.1).

## Results

113 patients with an acute ischemic stroke in the MCA territory were included in this study. Mean age was 69 ± 13 years, and 40% were female. Baseline data and clinical variables of the study population are displayed in Table 1.

**Table 1:** Characteristics of the study population

	Non-dysphagic	Dysphagic	*p*-value
*n* (%)	51 (45.1)	62 (54.9)	–
Mean age, years (SD)	68.2 (± 12.6)	69.7 (± 12.8)	0.553
Female, *n* (%)	21 (41.2)	24 (38.7)	0.470
Left hemispheric ischemia, *n* (%)	32 (62.8)	40 (64.5)	0.500
Median ASPECTS (IQR)	8 (6.0–9.0)	7 (5.0–8.0)	0.001*
Median NIHSS, (IQR)	6 (4.0–9.0)	13 (7.0–17.0)	0.000*
Aphasia, *n* (%)	20 (39.2)	39 (62.9)	0.010*
Mutism, *n* (%)	2 (3.9)	17 (27.4)	0.001*
Dysarthria, *n* (%)	28 (54.9)	31 (50.0)	0.371
Dysphonia, *n* (%)	13 (25.5)	20 (32.3)	0.282
Abnormal Gag Reflex, *n* (%)	5 (9.8)	23 (37.1)	0.001*
Wet voice, *n* (%)	3 (5.9)	3 (4.8)	0.564
Abnormal volitional cough, *n* (%)	10 (19.6)	14 (22.6)	0.441
Cough after swallow, *n* (%)	11 (21.6)	30 (48.4)	0.003*
Buccofacial apraxia, *n* (%)	8 (15.7)	31 (50.0)	0.000*

*SD* standard deviation, *IQR* interquartile range, *ASPECTS* Alberta Stroke Early CT Score, *NIHSS* National Institutes of Health Stroke Scale

*Bold values indicate significant difference (*p* < 0.05) between dysphagic and non-dysphagic patients for the respective item

According to FEES, 62 patients (54.9%) were classified as having dysphagia. Dysphagic patients more often had aphasia, were mutistic, had an abnormal gag reflex, and suffered from buccofacial apraxia than non-dysphagic patients. NIHSS at hospital admission was significantly higher in the dysphagic group as compared to the non-dysphagic group (Table 1).

**Fig. 1:**
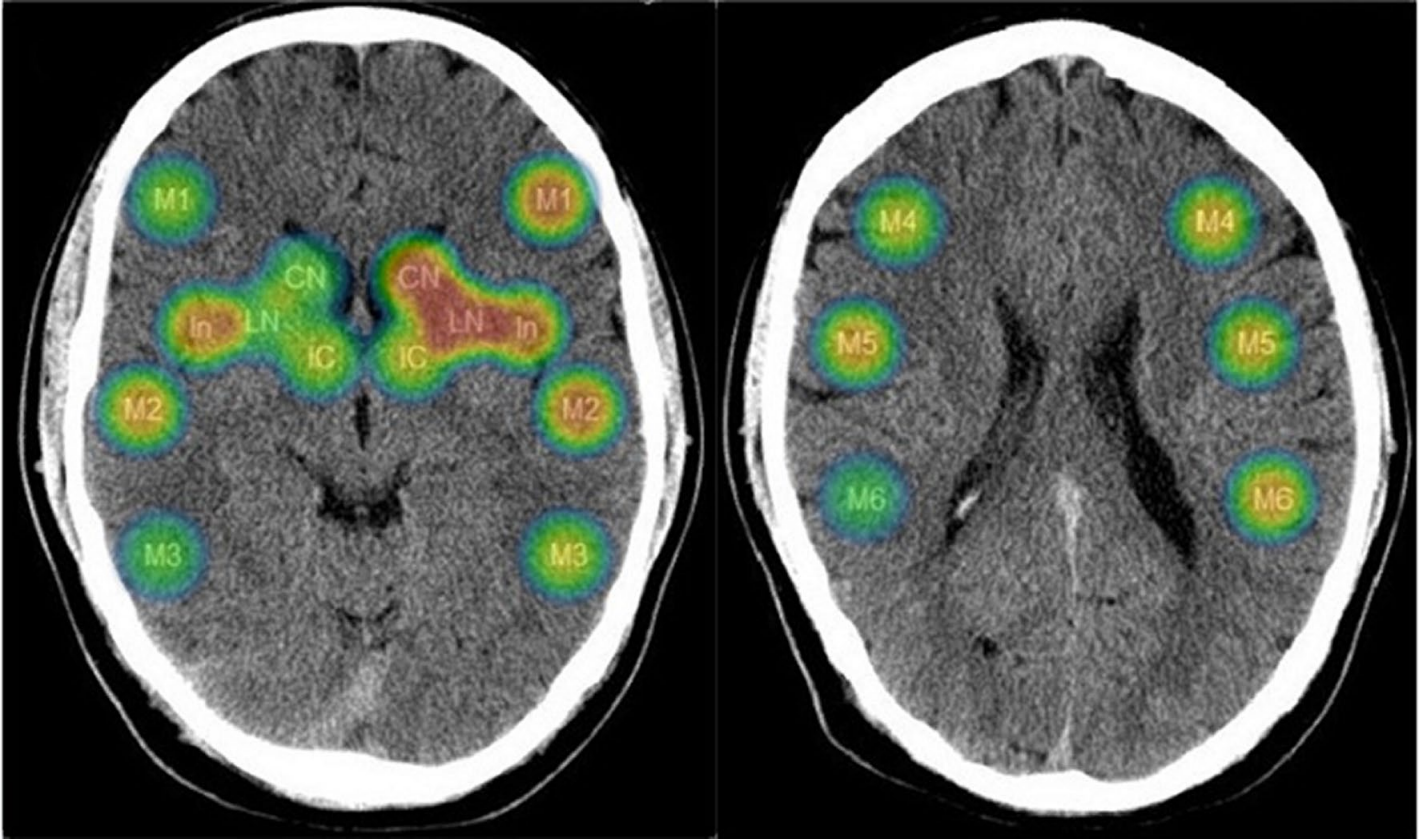
*Heat map* visualizing the association of affected ASPECTS segments (rated as “0”) with dysphagia. Dark blue color indicates low risk (OR 1.0) and dark red color indicates high risk (OR 0.1) for dysphagia. The “fading out” of the color in the peripheral zones is a design effect of the software tool. Color-coded odds ratio values lie between 1.0 and 0.1:

The frequency of dysphagia was not different between left- and right-sided MCA infarctions (40 out of 72 patients vs. 22 out of 41 patients; *p* = 0.500). Moreover, no difference in ASPECT sum scores was observed between strokes affecting the left or the right hemisphere (median 7 [IQR 5–9] vs. 7 [6–9]; *p* = 0.579). In the left hemisphere the strongest associations between the ASPECTS segments and dysphagia were found for the lentiform nucleus (LN) (OR 0.113 [CI 0.028–0.433; *p* = 0.001), the insula (In) (0.275 [0.102–0.742]; *p* = 0.011), and the frontal operculum (M1) (0.280 [0.094–0.834]; *p* = 0.022; see Table 2 and *heat map* [Fig. 1]). For the right hemisphere, the strongest association was found for the insula region (0.385 [0.107–1.384]; *p* = 0.144), however, without statistical significance. In a stepwise multivariate regression model including all ASPECTS segments of the left hemisphere, an independent association with dysphagia persisted for the lentiform nucleus (OR 0.089 [CI 0.021–0.379; *p* = 0.001) and the insula (OR 0.209 [CI 0.066–0.660; *p* = 0.008), respectively. No independent associations were found for the right hemisphere.

**Table 2:** Odds ratios for risk of dysphagia with respect to ischemic changes in brain regions according to ASPECTS, stratified for the left and the right hemisphere

	Left hemisphere	Right hemisphere
Caudate nucleus [CN], OR (CI)	0.152 (0.018–1.313) *p* = 0.087	0.907 (0.205–4.010) *p* = 0.897
Lentiform nucleus [LN], OR (CI)	0.113 (0.028–0.433) *p* = 0.001*	0.952 (0.238–3.811) *p* = 0.945
Internal capsule [IC], OR (CI)	0.641 (0.191–2.156) *p* = 0.472	0.711 (0.167–3.026) *p* = 0.644
Insula [In], OR (CI)	0.275 (0.102–0.742) *p* = 0.011*	0.385 (0.107–1.384) *p* = 0.144
Frontal operculum [M1], OR (CI)	0.280 (0.094–0.834) *p* = 0.022*	0.808 (0.220–2.964) *p* = 0.747
Anterior temporal lobe [M2], OR (CI)	0.296 (0.085–1.027) *p* = 0.055	0.467 (0.115–1.900) *p* = 0.287
Posterior temporal lobe [M3], OR (CI)	0.677 (0.179–2.563) *p* = 0.566	0.952 (0.238–3.811) *p* = 0.945
Anterior MCA territory immediately superior to M1 [M4], OR (CI)	0.540 (0.176–1.656) *p* = 0.281	0.667 (0.184–2.416) *p* = 0.537
Lateral MCA territory immediately superior to M2 [M5], OR (CI)	0.573 (0.221–1.485) *p* = 0.252	0.486 (0.139–1.704) *p* = 0.260
Posterior MCA territory immediately superior to M3 [M6], OR (CI)	0.490 (0.150–1.600) *p* = 0.237	1.021 (0.285–3.650) *p* = 0.975

*OR* indicates odds ratio, *CI* confidence interval

*Bold values indicate significant correlations (*p* < 0.05) between the affected ASPECTS segments and the presence of dysphagia

Table 3 shows the relative frequency of dysphagia rising up to 100% with respect to the number and location (“*hot spots*”, see Table 2) of affected areas in patients with left hemispheric strokes as illustrated by the *heat map*.

**Table 3:** Frequency of dysphagia with respect to the affected ischemic *“hot spots”* (lentiform nucleus, insula, and frontal operculum) in patients with left hemispheric strokes

Ischemic *“hot spots”*, alone or in combination	Total number of patients	Frequency of dysphagic patients	Median ASPECTS [IQR]	
Lentiform nucleus [LN] + Insula [In] + Frontal operculum [M1]	8	8 (100%)	4 [3.25–5]	
Lentiform nucleus [LN] + Insula [In]	12	12 (100%)	4.5 [3.25–6.75]	
Lentiform nucleus [LN] + Frontal operculum [M1]	8	8 (100%)	4 [3.25–5]	
Insula [In] + Frontal operculum [M1]	24	18 (75.0%)	5 [4–6]	
Lentiform nucleus [LN]	22	19 (86.4%)	7 [4–8]	
Insula [In]	37	26 (70.3%)	6 [4–7]	
Frontal operculum [M1]	24	18 (75.0%)	5 [4–6]	

*IQR* indicates interquartile range, *ASPECTS* Alberta Stroke Program Early CT Score

**Fig. 2:**
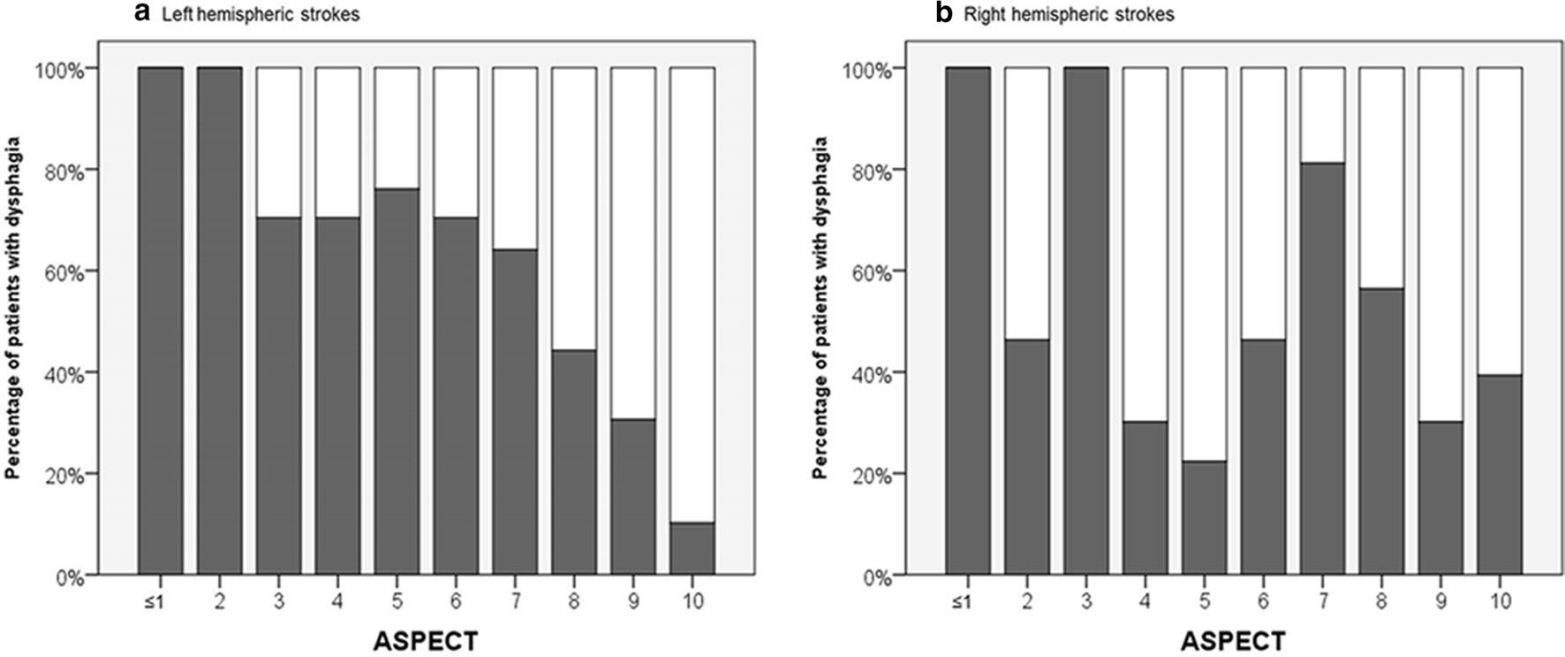
Bar chart visualizing the percentage of patients with dysphagia according to ASPECTS for left (**a**) and right (**b**) hemispheric strokes

ASPECT sum scores were lower in patients with dysphagia compared to patients without dysphagia (median 8 [IQR 6.0—9.0) vs. 7 [5.0–8.0]; *p* = 0.001; see Table 1). We found a steady increase of dysphagia risk with lower ASPECT scores for the left hemisphere. This was much less present in the right hemisphere (*bar chart*, Fig. 2).

Likewise, for unaffected “*hot spots*”, the risk of dysphagia increased with decreasing ASPECT scores; however, for affected “*hot spots*” a relevant risk of dysphagia already existed with higher ASPECT scores (see Suppl. Table 1a/b).

For the left hemisphere, multivariate logistic regression analysis including age and sex revealed lower ASPECT sum scores (OR 0.617 [CI 0.457–0.834]; *p* = 0.002) to be independently associated with dysphagia, whereas for the right hemisphere (0.738 [CI 0.528–1.032]; *p* = 0.076), this association was not present. Due to collinearity, the additional inclusion of the NIHSS score into the model abolished the predictive value of the ASPECT sum score in favor of the NIHSS (left hemisphere; OR for NIHSS 1.206 [CI 1.064–1.367]; *p* = 0.003).

The median *Fiberoptic Endoscopic Dysphagia Severity Scale* (FEDSS) in dysphagic patients was not different between the left and the right hemisphere (median 4.5 [IQR 4–5], *n* = 40 vs. 4.0 [2.75–5], *n* = 22; *p* = 0.578). We did not observe an association between ASPECTS segments and the severity of dysphagia.

A multivariate regression analysis including age and sex showed no significant association between lower ASPECTS score and pneumonia for both left hemispheric strokes (OR 0.802 [CI 0.626–1.028]; *p* = 0.081) and right hemispheric strokes (0.864 [CI 0.639–1.170]; *p* = 0.345). Moreover, a significant association between pneumonia and the three “*hot spot*” segments of the left hemisphere could not be observed.

## Discussion

The main finding of this study is that a standardized neuroanatomical lesion map based on the ASPECTS can be used in the acute phase of stroke to identify ischemic “*hot spot*” lesions associated with dysphagia. Our analysis revealed different associations between ASPECTS segments and dysphagia for both the left and the right hemisphere, thus providing interesting insights into the cortical and subcortical representation of swallowing within the MCA territory.

In the right hemisphere, only the involvement of the insular cortex tended to be associated with swallowing dysfunction. In contrast, involvement of the lentiform nucleus, the insular cortex and the frontal operculum in the left hemisphere were predictive for dysphagia. Our findings are in line with results from previous studies. The inferior precentral gyrus and the insular cortex are known to represent critical nodes of the supratentorial deglutition network [15, 23, 33]. The insular cortex has been associated with related swallowing and nutritional properties such as coordination of oral musculature gustation, and autonomic functions, through connectivity or inherent ability of its own [34]. Its general involvement in swallowing has been consistently demonstrated over many studies, while findings are diverse as to whether there is a right or left hemisphere lateralization of this region [19, 35, 36]. Several authors suggest that the left insula strongly contributes to the initiation of the swallow by processing sensory elements and interacting with widespread brain regions in both hemispheres [23, 37, 38]. Although not as pronounced as for the insula, a left-sided lateralization of the frontal operculum during swallowing has been reported [33, 39, 40]. The strong association between the lentiform nucleus and dysphagia within the left hemisphere needs to be highlighted as an interesting finding. Although data on the role of the lentiform nucleus as part of the basal ganglia are scarce, neuroimaging studies have confirmed its involvement in swallowing [22, 35, 41]. To what extend lentiform nucleus lesions contribute to the development of post-stroke dysphagia remains unclear. Our data attribute a more profound role of the left lentiform nucleus in swallowing.

Looking at the frequency of dysphagia in those patients with afflicted *“hot spot”* areas (Table 3) in the left hemisphere, the involvement of the lentiform nucleus solely was associated with a relative dysphagia frequency of about 80%. Considering a combination of the lentiform nucleus with either the frontal operculum and/or the insula increases the relative frequency of dysphagia up to 100%. Those patients with infarction involving one or more of these ASPECTS segments (frontal operculum, insular cortex, lentiform nucleus) should be targeted for a more profound evaluation by a SLP or instrumental swallowing assessment independently from swallowing screening result.

We observed an inverse correlation between dysphagia risk and ASPECT sum score, which was much stronger for the left than for the right hemisphere (Fig. 2). As a potential explanation, a more widespread representation of swallowing within the left hemisphere can be assumed. This is in accordance with available literature describing greater anatomical connectivity for swallowing in the left hemisphere [23, 42].

We enriched the analysis by the additional evaluation of pneumonia incidence in our patients. A trend towards an association between lower ASPECT scores and pneumonia was observed for the left hemisphere.

The severity of neurological deficits in terms of NIHSS has been identified as an important clinical predictor of post-stroke dysphagia [3, 43, 44]. For example, a NIHSS score of > 13 is used as a triage instrument to facilitate the choice of an alternative feeding route within 48 h after stroke [45]. However, NIHSS scoring underlies a hemispheric bias as it over-represents comprehension-dependent (left hemisphere-associated) tasks rather than tasks relying on right hemisphere functions [46, 47]. For example, imaging studies have shown that patients with right hemispheric stroke can have low NIHSS scores despite substantial infarct volumes [46]. Here, ASPECTS enables a more objective assessment of stroke magnitude. Consequently, no difference was found for the ASPECT sum score between left- and right-sided MCA strokes.

Strengths of our study are the prospective recruitment of a homogeneous collective of patients with MCA infarctions. We consistently used FEES which is considered to be an objective swallowing assessment tool that offers an excellent diagnostic accuracy and inter-rater reliability in acute stroke patients to evaluate the presence of dysphagia in all patients in a time window of 24 h after hospital admission [25, 26, 48].

Shortcomings include the limited number of patients, thereby leaving the statistical evaluation of the right MCA infarction group underpowered. Moreover, the results for the frontal operculum (M1) have to be interpreted with caution, since the statistically significance did not withstand after adjustment for multiple comparisons. In order to accurately detect the extent of ischemic changes in CT scans according to ASPECTS, we analyzed the first CT scan with clear ischemic changes. Therefore, our findings might be limited regarding CT brain imaging in the very early phase of stroke, where ischemic changes still might be missing. Moreover, brain imaging consisted of both CT and MR scans, which might have caused heterogeneity in the determination of the ASPECT score. However, previous data showed that the differences between CT and MR imaging (DWI) in visualizing early infarction are small when using ASPECTS [32].

## Conclusion

In summary, the distribution and extent of ischemic changes in the MCA territory according to ASPECTS may be used as risk indicator of neurogenic dysphagia in MCA infarction, particularly when the left hemisphere is affected. Although hitherto radiological findings are not frequently used in acute dysphagia management our data show that lesion location defined via ASPECTS provide additional predictive information. However, due to the exploratory nature of this research external validation studies of these findings are warranted in future. Nevertheless, we believe that this work might stimulate further research to transfer radiological parameters in acute dysphagia management.

## Electronic supplementary material

Below is the link to the electronic supplementary material.

Supplementary file1 (PDF 92 kb)

## Data Availability

Anonymized data used in this study are available on request from Dr. Sriramya Lapa.
