# Supplementary material for: Ischemic Lesion Location Based on the ASPECT Score for Risk Assessment of Neurogenic Dysphagia
Source: Dysphagia. 2020 Nov 6;36(5):882–90. doi: 10.1007/s00455-020-10204-0 (PMC8464570; doi:10.1007/s00455-020-10204-0)
Supplement: Supplementary file 1 — Supplementary file1 (PDF 92 kb) [file 455_2020_10204_MOESM1_ESM.pdf]

Article title: Ischemic lesion location based on the ASPECT score for risk assessment of neurogenic dysphagia; Journal: Dysphagia; Authors: Sriramya Lapa, Christian Foerch, Oliver C. Singer, Elke Hattingen and Sebastian Luger; affiliation/e-mail address of corr. author: Department of Neurology, Goethe University, Frankfurt am Main, Germany; Sebastian.Luger@kgu.de

Suppl. Table 1a

Frequency of dysphagic patients (n = dysphagic / total) with respect to the affected ischemic *"hot spots"* for each ASPECT score (10 – 0)

**ASPECTS**	**LN affected, n = dysphagic / total**	**LN not affected, n = dysphagic / total**	**In affected, n = dysphagic total**	**In not affected, n = dysphagic / total**	**M1 affected, n = dysphagic / total**	**M1 not affected, n = dysphagic / total**
**10**						
**9**	3 / 4	4 / 16	x	7 / 20	x	7 / 20
**8**	3 / 5	6 / 12	6 / 9	3 / 8	3 / 5	6 / 12
**7**	4 / 4	10 / 15	7 / 10	7 / 9	3 / 4	11 / 15
**6**	1 / 1	7 / 11	6 / 9	2 / 3	5 / 8	3 / 4
**5**	3 / 5	2 / 4	5 / 9	x	4 / 6	1 / 3
**4**	4 / 6	0 / 1	4 / 6	0 / 1	3 / 5	1 / 2
**3**	3 / 3	3 / 4	6 / 7	x	5 / 6	1 / 1
**2**	2 / 3	x	2 / 3	x	1 / 2	1 / 1
**1**	1 / 1	x	1 / 1	x	1 / 1	x
**0**	1 / 1	x	1 / 1	x	1 / 1	x

ASPECTS indicates Alberta Stroke Program Early CT Score; LN, lentiform nucleus; In, Insula; frontal operculum, M1. For cells marked with x there is no data available that meets the criteria.

Suppl. Table 1b

Frequency of dysphagic patients (%) with respect to the affected ischemic *"hot spots"* for each ASPECT score (10 – 0)

**ASPECTS**	LN affected, dysphagic in %	LN **not** affected, dysphagic in %	In affected, dysphagic in %	In **not** affected, dysphagic in %	M1 affected, dysphagic in %	M1 **not** affected, dysphagic in %
**10**						
**9**	75%	25%		35%		35%
**8**	60%	50%	67%	38%	60%	50%
**7**	100%	67%	70%	78%	75%	73%
**6**	100%	64%	67%	67%	63%	75%
**5**	60%	50%	56%		67%	33%
**4**	67%	0%	67%	0%	60%	50%
**3**	100%	75%	86%		83%	100%
**2**	67%		67%		50%	100%
**1**	100%		100%		100%	
**0**	100%		100%		100%	

ASPECTS indicates Alberta Stroke Program Early CT Score; LN, lentiform nucleus; In, Insula; frontal operculum, M1. On a continuous color scale dark green color indicates low frequency and dark red color indicates high frequency of dysphagia. For gray-shaded cells there is no data available that meets the criteria.
